# Thoracentesis

**Published:** 1874-04-01

**Authors:** 


### THORACENTESIS.

BOSTON SOCIETY FOR MEDICAL IMPROVEMENT, NOV. 24, 1873.

*From Boston Medical and Surgical Journal.*

DR. LYMAN reported a case to show the good results of this operation.

The patient, a man aged twenty-six, had a cough, with pain in his right side and dyspnœa, for about six weeks; and, latterly, there was some œdema of the feet. He was tapped, and is doing very well, showing a marked contrast to a patient in the next bed to him, who had gone on for a long time with an effusion, and who now has empyema. He also spoke of a case where he had made a permanent opening, and put in a tube. The opening had healed, and the patient was discharged, well, although there was considerable contraction.

Dr. Tarbell spoke of a case in which Dr. Minot had made a permanent opening, by cutting down between the ribs, which had done very well; also of a case in his own ward, where pus showed signs of pointing near the cardiac region. This was opened, and nine pints of pus removed. The patient, however, not doing as well as was expected, after exploring with the pneumatic aspirator, Dr. Tarbell cut into the pleural cavity in the usual place, and put in a tube. The improvement since has been very marked, the patient having grown fat and strong.

Dr. Bowditch said that he had always advocated free opening in these cases, but that at the present time the fact of the patient's insisting upon having ether would modify his treatment in some cases. He knew of four cases, within a year, where thoracentesis had resulted fatally, and he felt sure that ether had a great deal to do with the result.

Dr. Ellis said that those cases where an operation was indicated, and the use of an anæsthetic was to be feared, would seem to be proper ones for the use of the atomizer with rhigoline, or some other freezing agent.

Dr. Lyman said that he was very glad that Dr. Bowditch had called attention to the possible danger in the use of ether in such cases, and was convinced that his idea was perfectly just and well-founded. He had reason to think that if the case he had just reported had been etherized, he would have died from the fact of the extreme dyspnœa, sense of constriction, and faintness, which followed (causing him much uneasiness for fifteen minutes), and which was only overcome by prompt and repeated stimulation, and by encouragement of the patient to some personal effort, neither of which would have been available had he been paralyzed by ether. He farther said that the cause of the temporary distress was, doubtless, due to the removal of too much fluid at one sitting, the lung not being readily expansible, and the neighboring organs, especially the heart, being so suddenly dislocated to occupy the space from which the fluid had been removed.

Dr. Ellis asked Dr. Bowditch with regard to the indications for stopping in the operation, and whether the severity of the cough and the action of the pump were not the most reliable ones.

Dr. Bowditch said that a very severe fit of coughing would make him stop; but that he considered a moderate amount as rather a good sign, as it shows that the lung is expanding. What he considered the most important indication to cease drawing off the fluid was to be got from the feelings of the patient himself; namely, as soon as a sense of stricture or constriction across the chest was complained of, the operation should cease.

*December 8th.* — Dr. Tarbell showed the two cases of thoracentesis of which he had spoken at the last meeting, one of whom had been operated on by Dr. Minot, and the other by himself. They both were very satisfactory, the openings having healed, and percussion and auscultation showing that the lungs had returned to almost their normal condition. He then read the following paper:

"As the subject of empyema was brought up at the last meeting of this Society, and objections were raised to the etherization of any patient into whose thorax a free opening was to be made, I will report two cases which have lately been treated in the wards of the Massachusetts General Hospital, and will exhibit the patients.

"One case will show, as far as *one* case may, that ether is not necessarily unsafe in all cases of empyema. The other is a fair type of the cases where it is at least prudent not to give ether. Both cases will, I think, help to show that the danger lies, not in making a free opening while the patient is etherized, but in etherizing while the lungs are so oppressed that the least addition to their burden causes asphyxia.

"I.—Katy H., thirteen years old, a mill-operative, was admitted to the hospital June 20, 1873. Her mother died of some lung disease. Her father is living and healthy. She has always been well until the present attack, which began two weeks previously, without known cause, with a short, hacking cough, but no expectoration. The next day Dr. Minot examined her, and found the usual symptoms of considerable fluid in the left pleural cavity, the heart being so dislocated that its impulse was heard and seen directly at the xiphoid cartilage. The next day Dr. Minot performed paracentesis, removing about three pints of purulent fluid with the syringe. She seemed relieved by the operation; but the next day the pulse and respiration began to grow worse again, increasing in frequency until the 30th, when the record reads, 'pulse 135; respiration 48; temperature 104.2°; cough urgent.'

"The patient having been fully etherized, Dr. Minot made an incision into the left pleural cavity, between the eighth and ninth ribs, below the scapula, allowing the escape of about a quart of inodorous pus. He then inserted a small rubber drainage-tube, and syringed out the cavity with water.

"On the second day afterward the patient came into my charge, her pulse having gone down from 135 to 100, the respirations from 48 to 30 per minute, and the temperature from 104° to 99.5°.

"The cavity was thoroughly syringed out, twice daily, with a weak solution of carbolic acid. Under good nourishment, mild stimulants, and no medicine except an occasional opiate, she steadily improved. In two weeks after the operation she was carried out into the garden. In four weeks, she walked out. In four and one-half weeks the discharge was but a few drops daily, and the tube was removed. Its removal was premature, however, for in twenty-four hours she lost her appetite, became hot and feverish, with a pulse of 140, and temperature of 103°. The tube was replaced, the syringe practiced as before, and continued until October 17th, fifteen weeks after the operation, when the tube was finally removed. The wound healed in two weeks.

"At the time of the removal of the tube, the left side was much smaller than the right; but since then the lung and left side of the thorax have expanded, until they are now nearly as large as the right; and there is very little deformity. The heart has returned to its normal position. The respiratory murmur is good over the left front, and fair over the back, down to within about two inches of the cicatrix of the incision. The patient is fat and rosy, with good appetite and strength.

"II.—Manuel Antone, twenty-one, single, a Portuguese laborer, ignorant of our language, was brought to the hospital July 27, 1873, without any history except that he had been sick two months with cough, sharp pain in the left side, and, latterly, urgent dyspnœa.

"On examination, I found the symptoms of enormous distention of the left side of the thorax with fluid, which forced itself out between the ribs at some points, and was burrowing beneath and among the muscles in front of the chest. The heart was dislocated so far that its apex-beat was visible to the right of the sternum. He was literally gasping for breath.

"Without etherizing him, I made an incision through the skin and muscles, just below and to the outside of the left nipple, giving exit to four and a-half quarts of creamy, inodorous pus. The distention was so great that the pus was at first expelled in a large stream to a distance of ten or twelve inches from his body. He immediately expressed a sense of relief. A poultice was applied to the wound, and frequently changed. Pus followed in large quantities from the incision, and the patient felt a little easier; but there were no means of determining the location of the perforation of the pleural cavity; and the serious symptoms did not abate. The pulse continued about 120, the respiration 34, and the temperature 102°.

"After waiting three days, I made another incision, plunging the knife directly into the left pleural cavity, between the ninth and tenth ribs, about four inches below and behind the angle of the scapula, giving exit to about two quarts of pus. A small rubber drainage-tube was introduced. The cavity was thoroughly syringed out twice daily with a weak solution of carbolic acid (twelve grains to the pint of water). Little or no pus came from the first incision after the drainage-tube was introduced. Stimulants and nourishing food were all the medicine he had.

"He improved steadily, with the exception of a single occasion when the tube became misplaced. In three weeks he began to sit up. In four weeks he was carried out into the garden. The discharge decreased slowly in amount until November 18th — sixteen weeks after opening the thorax—when the tube was finally removed. The wound quickly healed. The heart has returned nearly to its normal position. There is resonnance over the left side down to a line drawn about one inch below the angle of the scapula, and respiration is heard nearly to this line. There is but little deformity, and there is no lateral deviation of spine.

"I think the successful results in these two cases are due to the persistence with which the wounds were kept open, and to the free drainage permitted by the tube, and also to the faithful and thorough daily syringing of the cavity, carried out by Mr. Rotch, the house-officer.

"I have here a very efficient, and at the same time simple, contrivance for retaining the drainage-tube securely. It was invented by Mr. Mosely, one of the present house-officers at the hospital. It is a rubber diaphragm, about one and one-half inches in diameter, and a strip of tin two inches wide, through the center of which the tube passes, and to which it is easily made fast by adhesive plaster. The ends of the strip of tin are then bent sharply upward; the tube is inserted into the wound, the convex surface of the tin turned toward the thoracic walls, to which the ends are made fast by adhesive plaster, thus gently pressing the diaphragm against the wound, holding the tube firmly in place, and at the same time preventing leakage around it.

"To make the tin spring for retaining the drainage-tube in its place, take a strip of common sheet tin, one inch wide and ten inches long. In the middle of this make a hole just large enough to admit, but not constrict, the tube to be used. At a distance of three-quarters of an inch from this hole, on each side, bend the tin at a right angle, so as to form a letter U. At one inch from one of these right angles bend the tin at another right angle, in an opposite direction to the first. Pass a narrow strip of adhesive plaster around the U-shaped depression, so as to prevent its spreading. The tube is to be passed through the hole in the bottom of the U-shaped depression, which is to press against the diaphragm through which the tube passes. Pad the ends of the spring with cotton-batting, bend it to fit the form of the person to whom it is to be applied, and attach it with adhesive plaster.

"It was suggested at the last meeting that the symptom of a sense of constriction across the thorax, which is relied on as a warning of approaching danger, would be wanting if the patient were etherized. I believe it is conceded that this symptom is induced by the creation of a partial vacuum in the cavity of the thorax, which the lung cannot expand to fill. If a free opening is made with the knife, the air rushes in to replace the fluid flowing out—no vacuum is formed, and this objection does not hold.

"I believe, also, that it is not the usual custom — it certainly is not at the hospital — to etherize for the removal of fluid by means of Dr. Wyman's suction-syringe, or aspirator.

"Of these two cases, the first was fully etherized; and she suffered no ill effects from the ether at the time of the operation, nor subsequently. The second was not etherized. The idea of using ether did not occur to me, nor was it suggested by any of the physicians or surgeons present. The patient seemed to be having as much as he could do to get breath, even aided by voluntary efforts; and it would have taken but a slight addition to the burden to have stopped his breathing altogether. A little more pus would have done it. Had I etherized him, and in doing so deprived him of the little oxygen he was getting, the ether would have been exactly as important an element in causing his death as the proverbial 'last straw' was 'in breaking the camel's back.' The previous load must be taken into account, and carefully estimated. It seems to have been pretty well demonstrated that a patient may be asphyxiated while being etherized for any operation.

"The additional danger in etherizing these cases lies in the fact that there is much less lung tissue in working-order, through which the system may obtain the necessary oxygen; but the danger is immediate; and I do not see how ether can be justly accused as the cause of death occuring ten days, or four days, after the operation. The idea of the knife plunged directly into the thoracic cavity, and the pain also, is something terrible, and we would all wish to save our patients from such agony, if possible. I believe that many of these cases may be *safely* etherized, if it is slowly and carefully done."

Dr. Bowditch said that these cases show how surgical interference at the right moment may save life. He was still, however, of the same opinion as at the previous meeting, namely, that in cases where the effusion is so great as to seriously interfere with the respiratory function, ether should be given with great caution.

Dr. Ellis showed two patients, to illustrate the curved line of dullness in cases of pleuritic effusions, of which he had previously spoken. One of the patients had been tapped; in the other, the absorption had been spontaneous. Both showed, in a very marked manner, that the highest point of dullness was at the side, the line gradually falling as it approached the spine. This line was mapped out by the percussion so as to be evident from any part of the room. The respiration, he stated, also followed this line.

Dr. Minot spoke of one point, in connection with the case shown by Dr. Tarbell, in which a permanent opening had been made, namely, the very great advantage of syringing the pleural cavity out with a solution of carbolic acid. This so effectually controls the bad odor, which is always present in these cases, that, by this means, the operation can be done in a large ward, instead of having to isolate the patient as was formerly the case.

